# Editorial: New lines of inquiry for investigating visual search behavior in human movement

**DOI:** 10.3389/fpsyg.2023.1145859

**Published:** 2023-02-22

**Authors:** Matthew A. Timmis, Matt Miller-Dicks, Alessandro Piras, Kjell Van Paridon

**Affiliations:** ^1^Cambridge Centre for Sport and Exercise Sciences (CCSES), School of Psychology and Sport Science, Anglia Ruskin University, Cambridge, United Kingdom; ^2^School of Sport, Health and Exercise Science, University of Portsmouth, Portsmouth, United Kingdom; ^3^Department of Neuromotor and Biomedical Sciences, University of Bologna, Bologna, Italy

**Keywords:** visual search, *Quiet Eye* (QE), virtual reality, visual blur, eye movements, head movement

The accurate and skilful execution of human movement requires allocating attention toward task-relevant information. Directing attention to pertinent areas within the environment is captured through measuring an individual's gaze behavior, with measures traditionally focusing on the locations and durations of the individual's gaze (summary fixation data). Understanding an individual's visual search behavior has provided important information regarding the link between oculomotor control, visual perception and attention allocation in task execution. The development of this understanding can originate from examining the inter-individual differences in visual search behavior as a function of expertise, or exploring intra-individual differences between successful and unsuccessful mastery attempts (e.g., Brams et al., 2019). Within this Research Topic, Kassem et al., examines the critical fixation points and durations associated with superior decision-making within an elite group of Australian Rules football players. Whilst watching game-based video clips players were asked to identify “the best option” in the developing play. Findings support previous conclusions (e.g., Vaeyens et al., 2007; Mann et al., 2019) that more experienced (skilful) players decide earlier, more correctly and use more task relevant information to inform their decision. These results also highlight how, within an elite (professional) environment, decision-making can be objectively assessed to more accurately distinguish the skill levels between players, thus providing a reminder of the potential value of video-based research to develop and enhance decision-making skills in elite/professional athletes (e.g., Milazzo et al., 2016).

Shinkai et al., investigate the role that both eye and head movements independently play when acquiring visual information during table tennis rallies between different competition levels. During rallies, the more expert players tended to keep watching the ball for shorter time than the semi-expert players, exhibited a larger gaze-ball angle (difference between gaze position coordinate and ball position coordinate—similarly observed by Mann et al., 2013) and slower eye velocities. There was no difference in head velocities between groups. Whilst it is understood that in gaze control, eye and head movements interact with each other, Shinkai et al., demonstrate that gaze angle and eye movements during table tennis rallies were associated with eye movements rather than head movement.

In addition to the dependent variables outlined in the work produced by Kassem et al., and Shinkai et al., the *Quiet Eye* is a variable commonly used to understand how skilled performers regulate action—final fixation on an object or target before the onset of a goal directed movement (Vickers, 1996). Where typical analysis of *Quiet Eye* has tended to assume the group mean data is reflective of all trials, variability around the mean may in fact show how the *Quiet Eye* is a flexible and adaptive perceptual tool (e.g., Ramsey et al., 2020). To illustrate the adaptive variability in the *Quiet Eye*, Franks et al., produce a descriptive case study of football (soccer) goalkeepers during a 1 vs. 1 (attacker vs. goalkeeper) scenario. *Quiet Eye* of professional football goalkeepers was analyzed as a shooter (opponent) dribbled with the ball 20 m from the goal line and shot the ball when reaching a pre-defined line at 11 m. Results highlight that the typical use of mean values to describe the *Quiet Eye* was not necessarily a prominent characteristic of successful performance. This is partly explained through interactions between the goalkeeper, their opponent and the environment, changing trial-to-trial, which may mean an information source in one trial may not emerge or may not be a successful determinant in the next trial with *Quiet Eye* patterning emerging through the performer-environment relationship (as proposed by Dicks et al., 2017).

Virtual reality (VR) technology provides an alternative method of understanding perception-action coupling (e.g., Craig et al., 2011). Technological advancements are such that they provide a viewing perspective which aims to replicate that of the expertise domain; enabling a viewer to acquire depth cues, experience first-person viewpoint and undertake visual exploration which utilizes head and eye movements. The study by Vu et al., utilizes a VR head-mounted display to explore visual tracking performance and underlying gaze activity during a team soccer task. Participants (soccer players and non-soccer players) assumed the vantage point of a central defender on a soccer field and visualized situations with 10 virtual moving teammates and 11 opponents, where they were required to track 4 “target players.” Visual tracking performance was higher in soccer players than in non-soccer players regardless of whether tracking players moving in soccer-specific trajectories or pseudo-random trajectories. Differences in gaze activity between groups of participants varied according to the spatial distribution of the virtual players, which also affected visual tracking performance. There was some indication that using soccer-specific trajectories might not be sufficient to replicate the representativeness of the “field” conditions with future work to consider increasing saliency of player's discernible features such as postural cues.

With previous research demonstrating the limited impact blur has in various sporting tasks Limballe et al., use a VR head-mounted display to specifically manipulate either central or peripheral locations when an expert combat athlete was required to intercept (block) an opponent's incoming punch. Similar to previous research (e.g., Mann et al., 2010), Limballe et al. demonstrate that expert combat athletes did not deteriorate significantly following a gaze-contingent blur manipulation. The only significant difference was between the central blur and peripheral blur conditions. Gaze data was predominantly located on the head, trunk and distal ends of the opponents two arms, similarly found in previous martial arts studies (e.g., Ripoll et al., 1995). Participants also demonstrated a reduction in the number and length of fixations from the first to second, and second to third punch, which may have been attributed to experts being better able to anticipate an attacking sequence once it had begun.

The goal of this Research Topic was to examine the emerging approaches to understanding the role of visual search in human movement. The varying aspects covered in this Research Topic highlights the continued growing interest in understanding visual search behavior in human movement and the articles within the topic provide insightful ideas for continuing to develop future research.

## Author contributions

All authors listed have made a substantial, direct, and intellectual contribution to the work and approved it for publication.
